# Differences in HIV infection trends in two regions of Cameroon with a longstanding HIV epidemic: insights from 2012 and 2022

**DOI:** 10.3389/fpubh.2025.1517213

**Published:** 2025-02-12

**Authors:** Yannick F. Ngoume, Urmes C. Teagho, Brice Eselacha, Oumarou H. Goni, Dell-Dylan Kenfack, Mérimé Tchakoute, Georges Nguefack-Tsague, Marcel Tongo

**Affiliations:** ^1^Centre of Research for Emerging and Re-Emerging Diseases/Institute of Medical Research and Studies of Medicinal Plants (CREMER/IMPM), Yaoundé, Cameroon; ^2^Ministry of Scientific Research and InnovationNational Institute of Cartography (INC), Yaoundé, Cameroon; ^3^Programmes de Santé et Développement au Sein du Groupement de la Filière Bois du Cameroun (GFBC), Yaoundé, Cameroon; ^4^Faculty of Medicine and Biomedical Sciences, The University of Yaoundé I, Yaoundé, Cameroon; ^5^HIV Pathogenesis Programme, The Doris Duke Medical Research Institute, University of KwaZulu Natal, Durban, South Africa

**Keywords:** HIV prevalence, Cameroon, HIV diagnosis, HIV diversity, HIV infection

## Abstract

**Introduction:**

To achieve the UNAIDS 95-95-95 target by 2025, it is of great importance to test and diagnose individuals infected with HIV; especially those residing in communities with limited access to health and in areas with a longstanding HIV epidemic, where the virus has been circulating since the early phase of the pandemic. In this regard, we determined the HIV prevalence in remote communities of the East and South administrative regions of Cameroon where the four cross-species SIV transmissions that gave rise to the four HIV-1 groups likely happened.

**Methods:**

We did this in two different periods: 2012/2013 (Period 1; 4,435 participants enrolled in the East and 2,347 in the South) and 2021/2022 (Period 2; 2,203 participant enrolled in the East and 2,347 in the South) using HIV1/2 rapid assays and standard ELISAs and according to the WHO testing strategy.

**Results:**

During Period 1, the HIV seroprevalence in the East was 6.9%. This prevalence had significantly decreased to 2.7% [Prevalence Difference or PD: 4.1 (3.1; 5.2); *p* < 0.001] by Period 2. Contrasting with these results, the HIV prevalence in the South during Period 1 was 5.5% and did not significantly change by Period 2 at 5.2% [PD: 0.3 (1.07;1.6); *p* = 0.67].

**Conclusion:**

Our data suggest that HIV transmission in remote communities of the South administrative region of Cameroon has likely remained uncurbed over the past decade. As a result, this region should be prioritized in efforts to curb the spread of HIV and reduce its prevalence.

## Introduction

The scaling-up of antiretroviral therapy (ART) in Cameroon, as with most countries in Africa, has likely contributed to the decline of the national prevalence of HIV-1. Yet the prevalence of circulating viruses in the country remains heterogeneously distributed within different Cameroonian communities. In 2018, Cameroon’s 2.7% HIV prevalence was amongst the highest in West-Central Africa (1). The Cameroonian HIV epidemic varies greatly across the country with the Adamawa and South administrative regions having the highest estimated prevalence (6 and 6.9% respectively) and the North-West region the lowest (2.1%) (2). According to UNAIDS, there were 14,000 new infections from all ages in the country and prevalence of HIV among young women and men was below 2% (3). In 2020, UNAIDS set a new ambitious target of 95–95–95 to help end the AIDS epidemic, whereby, by 2025, we should have diagnosed, treated and suppressed the viral loads in 95% of people living with HIV. Despite the tremendous gains that this program has achieved, we are still far from ending the epidemic. Diagnostic challenges and inequalities in treatment coverage are the most significant obstacle to achieving this target (4).

There are four groups of HIV-1 (group M, N, O and P), each originated from a cross-species transmission from chimpanzee or gorilla into human; and these events likely happened in locations that encompassed the East and South administrative regions of Cameroon (5). While three of these events resulted in limited spread within humans, one transfer gave rise to HIV-1 group M (HIV-1M) which is today responsible for the global pandemic (5). As a consequence of this, Cameroon has one of the most genetically diverse HIV epidemics in the world. Numerous different subtypes, circulating recombinant forms (CRFs), and unique recombinant forms (URFs) and other difficult to classify genetic variants in both urban and rural areas are found in the country (4). Furthermore, characterising viruses from these two administrative regions in Cameroon, has suggested that contemporary descendants of lineages present in the early phase of the pandemic are still circulating (6–8). This further supports the hypothesis that the epidemic in these two regions is likely among the oldest and most mature globally.

One key element of the strategy to eradicate AIDS is to test and diagnose individuals infected with HIV; especially in key populations and communities with limited access to healthcare systems, such as those residing in the equatorial rain forests of Cameroon where the virus has been circulating since it first jumped into humans during the early 20th century. Besides the low-resolution epidemiological data that has been used to identify wide geographical variations in HIV prevalence in urban settings, the actual seroprevalence of HIV across communities in rural equatorial rain forest regions of Cameroon are still unknown. Such information will be useful in the development of targeted control programs in these areas.

## Methods

### Villages and study population

**Figure 1:**
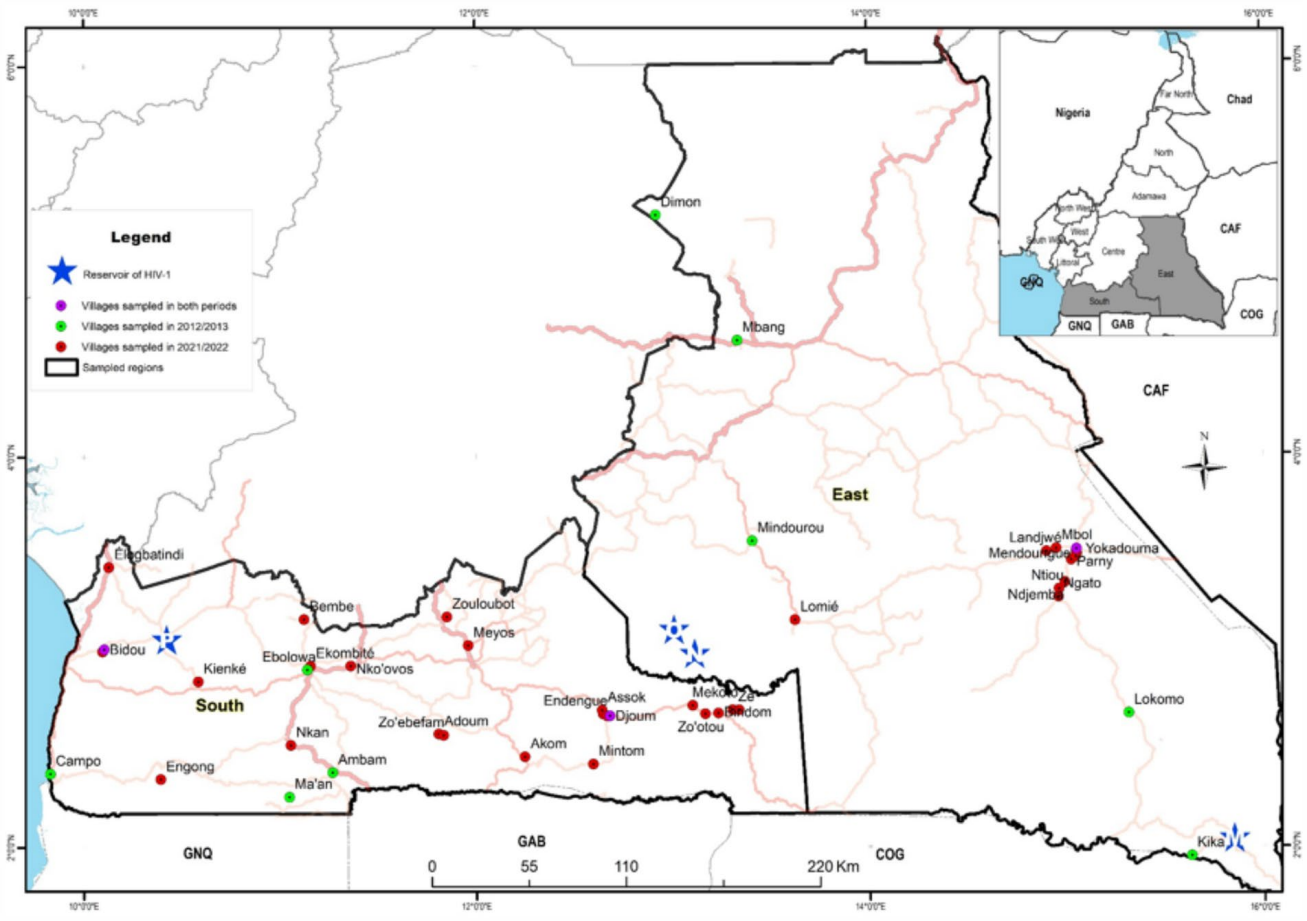
Map of the two equatorial rain forest regions of Cameroon illustrating the sites where samples were collected: red circles represent villages sampled in 2021/2022; green circles represent villages sampled in 2012/2013 and pink circles, villages sampled in the two periods. Blue stars represent the sites where HIV-1 group M (M), group N (N), group O (O) and group P (P) reservoirs were identified.

We conducted two health campaigns in 2012/2013 and in 2021/2022, in remote communities of the East and South administrative regions of Cameroon where reservoirs of all the major HIV-1 groups were found (groups M, N, and O in the East and group P in the South; Figure 1). The timepoints were informed by the national policy guidelines. The country implemented the ‘universal test and treat’ policy in 2016, to enhance progress towards the 95-95-95 global targets to end the epidemic by 2030 (9, 10). These campaigns were focused on general health issues (sexually transmitted diseases, high blood pressure, malaria, and food safety) and included voluntary HIV testing. Inclusion criteria comprised individuals with no obvious comorbidities above the age of 18 years willing to know their HIV status (i.e., the exclusion criteria involved any individuals with obvious comorbidities, who were unwilling to know their HIV status and/or who were under the age of 18 years). The health campaign began with a door-to-door awareness phase in each village, done by local health community workers. This phase lasted for about 5 days. Then, a mobile clinic was set up in a public place of the village for the next 2 days. Healthy individuals willing to know their HIV status were enrolled in the study. While the first campaign was entirely anonymous, demographic and behavioral information via a face-to-face interview using a structured questionnaire was collected in the second campaign. During the two campaigns, the procedure included a pre- and post-counseling as required by national Cameroonian guidelines (11). Blood was collected after the pre-counseling for HIV testing. Surveys were conducted in conjunction with local public health personnel who also ensured that individuals diagnosed with HIV were referred to the nearest healthcare centre for access to antiretroviral treatment according to national guidelines and procedures (11). Five ml of blood was drawn from all participants for HIV testing.

### HIV testing

**Figure 2:**
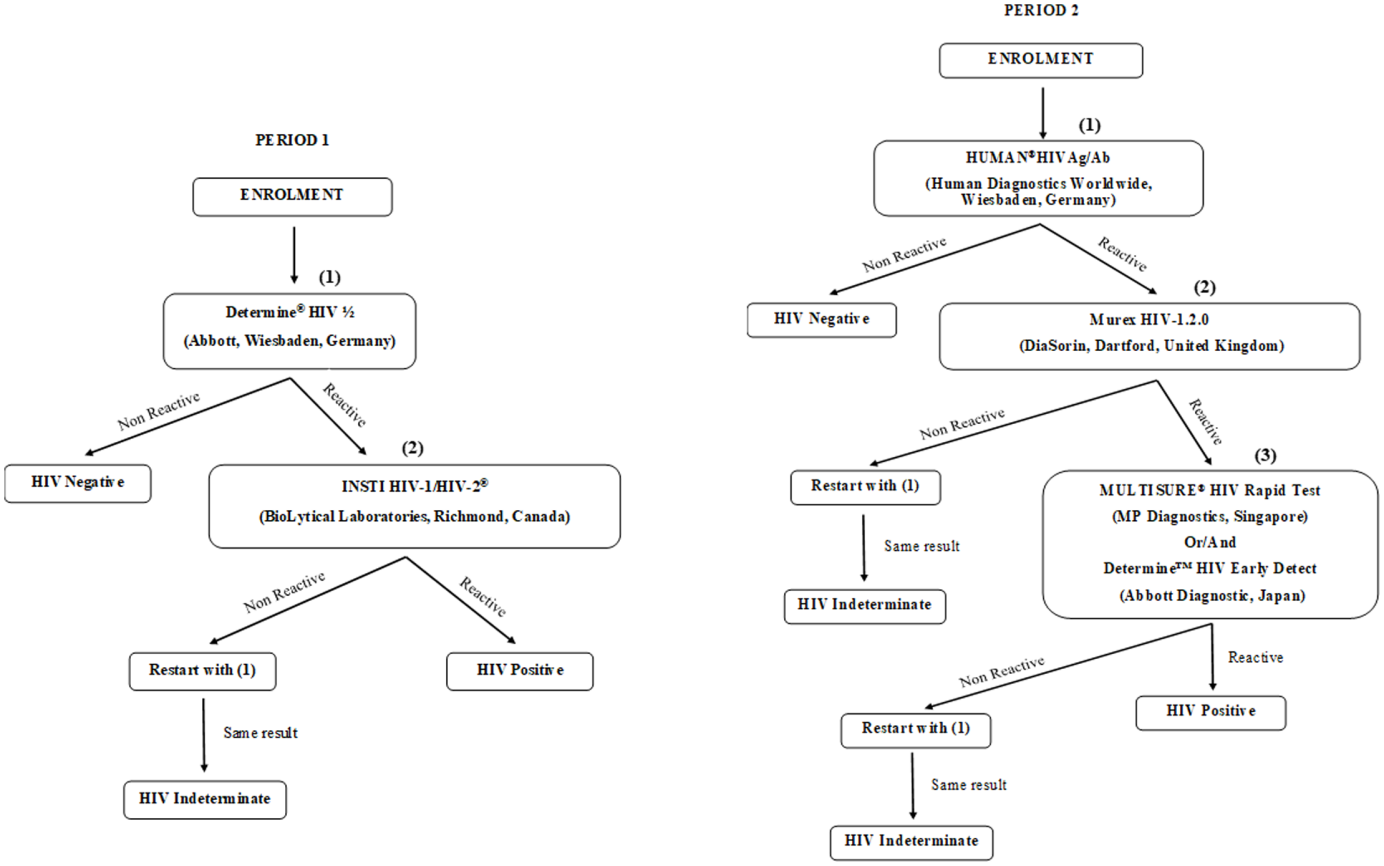
Flow diagram detailing the HIV testing strategy.

In the initial survey, serologic screening for HIV infection was conducted on-site using two point-of-care rapid tests that detect HIV antibodies (IgG, IgM, and IgA). The Abbott Determine HIV-1/2 rapid assay (Abbott, Wiesbaden, Germany) was used to pre-screen samples, with non-reactive results considered HIV-negative. Reactive samples (potentially HIV-positive) were then retested using the INSTI HIV-1/HIV-2 antibody test (BioLytical Laboratories, Richmond, Canada). Samples that tested positive on both assays were classified as HIV-positive. Discordant results (reactive on the first test but non-reactive on the second) were retested, and if the result remained the same, the participant was deemed “indeterminate” and advised to retest at a local health center after 1 month, while refraining from any risky behaviour. Data from participants with indeterminate results were excluded from further analysis (Figure 2).

In the second campaign, blood samples were transported to a specialized HIV-testing laboratory, where they were tested for HIV antibodies. Four different tests were conducted: two ELISA tests, the HUMAN®HIVAg/Ab (HUMAN Diagnostics Worldwide, Wiesbaden, Germany) and the Murex HIV-1.2.0 ELISA (DiaSorin, Dartford, United Kingdom), along with two rapid tests, the Determine™ HIV Early Detect (Abbott Diagnostic Medical Co. Ltd., Matsudo, Japan) and the MULTISURE® HIV Rapid Test (MP Diagnostics, Asia Pacific, Singapore). Plasma samples that were reactive in at least three of the tests were classified as HIV-positive. During Period 1, screening for HIV antibodies resulted in 378 “indeterminate” cases, while in Period 2, there were 41 “indeterminate” results. These indeterminate cases were excluded from further analysis (Figure 2).

### Statistical analyses

Data were analysed using STATA version 18. Frequencies and percentages (%) were used to describe qualitative variables. Pearson’s Chi-square test was used to establish relationships between qualitative variables. Associations were further quantified using unadjusted prevalence ratio (PR) and prevalence difference (PD) for univariable (bivariate) analysis and adjusted prevalence ratio (aPR) and adjusted prevalence difference (aPD) for multiple Poisson regression analysis (with interactions) with 95% confidence interval (CI), as recommended by Barros and Hirakata (12). Variables used for adjustment included administrative region and its interaction with the sampling period. Variables with *p*-values less than 0.05 were considered statistically significant.

The study was approved by the National Ethics Committee of the Cameroonian Ministry of Health (N° 2022/12/1510/CE/CNERSH/SP).

## Results

We visited six villages in 2012/2013 (Period 1) both in the East and South administrative regions of Cameroon; during this period, 4,425 participants were enrolled in the East, and 2,347 participants in the South. In the second health campaign performed in 2021/2022 (Period 2), we visited 11 villages in the East and 22 in the South, respectively, enrolling 2,203 and 2,062 participants. Only three villages were visited during both surveys: Djoum and Bidou in the South region and Yokadouma Rural in the East. Villages were selected based on the known acceptability of campaigns to their inhabitants and the availability of local staff.

While no socio-demographic data were available during Period 1, a total of 3,510 (1,687 in the East and 1,823 in the South) participants disclosed their sex information during Period 2; among them, 38% were female and 62% male. Across the region, these proportions were similar. The mean age was 36 years; greater in the South (40 years) than in the East (32 years). Table 1 summarises other socio-demographic data including marital status, education level, history of HIV testing and sexually transmitted diseases; overall these data were similar between the two regions.

**Table 1:** Characteristics of study individuals.

	East [*n* (%)]	South [*n* (%)]
Sex^*^		
Male	1,010 (59.9)	1,153 (63.2)
Female	677 (40.1)	670 (36.8)
Total	1,687 (100.0)	1,823 (100.0)
Age^*^ (Mean age 35.9, range: 18–101; years)		
18–21	433 (25.7)	125 (6.8)
22–31	536 (31.8)	503 (27.5)
32–41	426 (25.2)	483 (26.4)
42–51	165 (9.8)	339 (18.5)
52–61	81 (4.8)	195 (10.7)
62 or more	46 (2.7)	185 (10.1)
Education^*^		
No education	32 (2.4)	91 (7.5)
Primary studies	470 (35.2)	480 (39.3)
Secondary studies	715 (53.6)	590 (48.3)
Graduate studies	117 (8.8)	60 (4.9)
Marital Status^*^		
Single	531 (32.9)	408 (30.0)
Married (legal or not)	1,058 (65.5)	882 (64.8)
Divorced/Separated /Widow(er)	13 (0.8)	13 (0.9)
Widow (er)	13 (0.8)	59 (4.3)
History of HIV Testing^*^		
No	1,421 (97.1)	807 (89.5)
Yes	43 (2.9)	95 (10.5)
History of Sexual Transmissible Diseases^*^		
No	1,488 (88.2)	1,686 (92.1)
Yes	199 (11.8)	144 (7.9)

* Individuals who disclosed the information.

The multivariate Poisson model (Table 2) using period, region, and their interactions showed that, in terms of HIV prevalence, there was a significant interaction between geographical region and sampling period (adjusted Prevalence Ratio or aPR: 2.4 (1.6; 3.5); *p* < 0.001 and adjusted Prevalence Difference or aPD: 0.88 (0.5;1.3); *p* < 0.001). In addition, HIV prevalence showed significant (*p* < 0.001) disparities by region (aPR: 0.5 (0.38; 0.71); *p* < 0.001 and aDR: −0.7 (−1.0; −0.3); *p* < 0.001). This significant interaction implies that results should not be combined and must be presented per region.

**Table 2:** adjusted Prevalence Ratio (aPR) and adjusted Prevalence Difference (aPD) for multiple Poisson regression analysis (with interactions).

Parameter	aPD (95%CI)	aPR (95%CI)	*p*-value
Intercept	−2.9 (−3.1; −2.8)	0.05 (0.04; 0.06)	<0.001
Region^*^	−0.7 (−1.0; −0.3)	0.5 (0.38; 0.71)	<0.001
Period^**^	0.05 (−0.2; 0.3)	1.06 (0.8; 1.4)	0.680
Region*Period	0.88 (0.5; 1.3)	2.4 (1.6; 3.5)	<0.001

* Ref = South.

** Ref = Period 2.

**Figure 3:**
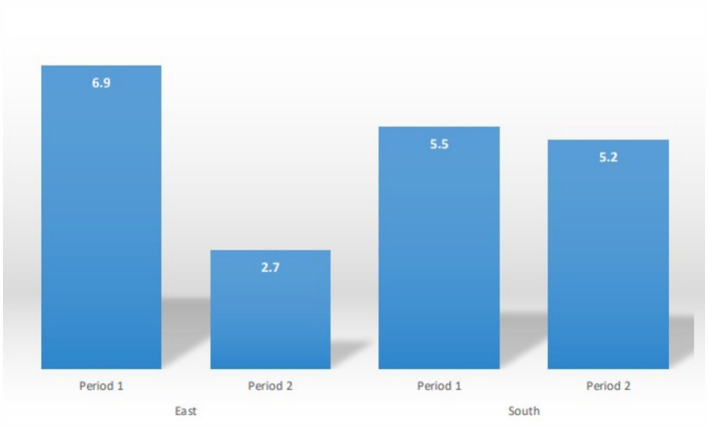
HIV Prevalence (%) per period and by region.

The stratified analysis (Table 3) reveals a decrease in HIV prevalence in the East between the two study periods, while the prevalence remained unchanged in the South. Specifically, during Period 1, 290 out of 4,223 samples from the East tested HIV-positive, resulting in a seroprevalence of 6.9%. This prevalence had significantly decreased to 2.7% [Prevalence Difference or PD: 4.1 (3.1; 5.2); *p* < 0.001] by Period 2 (Figure 3; Table 3). In contrast, 120 out of 2,171 samples from the South tested HIV-positive during Period 1, resulting in a prevalence of 5.5%. This prevalence remained relatively unchanged by Period 2, with a slight decrease to 5.2% [PD: 0.3 (1.07; 1.6); *p* = 0.67] (Figure 3; Table 3). Trends of HIV prevalence in the three communities that were visited during both the Period 1 and Period 2 campaigns revealed that the prevalence remained unchanged in Bidou (South) at 3.7% for both periods; while non significantly decreased in Djoum (South) from 5.3 to 4.0% (*p* = 0.34). A significant decline of the prevalence was observed in Yokadouma Rural (East) from 7.6% in Period 1 to 2.2% in Period 2 (*p* < 0.001).

**Table 3:** Stratified HIV prevalence per region and sampling period.

Region	Period	Negative [*n* (%)]	Positive [*n* (%)]	*n* (%)	PR (95%CI)	PD (95%CI)	*p*-value
East	1	3,933 (93.1)	290 (6.9)	4,223 (100.0)	2.5 (1.9; 3.3)	4.1 (3.1; 5.2)	<0.001
	2	2,120 (97.3)	59 (2,7)	2,179 (100.0)	1		
South	1	2,051 (94.5)	120 (5,5)	2,171 (100.0)	1.06 (0.8; 1.4)	0.3 (−1.07;1.6)	0.67
	2	1,938 (94.8)	107 (5.2)	2,045 (100.0)	1		
Total	1	5,984 (93.6)	410 (6.4)	6,394 (100.0)	1.6 (1.4; 1.9)	2.5 (1.6;3.3)	<0.001
	2	4,058 (96.1)	166 (3.9)	4,224 (100.0)			

## Discussion

The current analysis highlights differences in HIV infection trends over the decade from 2012 to 2022 in remote communities across two administrative regions of the equatorial rainforest in Cameroon, a country where HIV-1M has likely been circulating since the onset of the global HIV-1M pandemic. Our findings reveal a significant decline in HIV prevalence in the East, from 6.9 to 2.7%, over this period. In contrast, no similar decline was observed in the South, where the prevalence remained stable at around 5%.

Previous studies have also examined HIV prevalence in remote areas of the equatorial forest in Cameroon. In 2000, Nyambi and colleagues conducted a survey in locations including both the East and South regions of the country (13). They reported an HIV prevalence of 4.5% in the South and 6.9% in the East, which closely aligns with the data we obtained during Period 1. In a similar study, Edoul and collaborators investigated the prevalence of circulating HIV in three remote villages of Cameroon including two in the East and one in the South during the years 2011–2013 (14). They found that HIV was circulating at a prevalence of 9% in Moloundou/Mambele and 7% in Messok in the East (that gave an average of 8%); and 5.4% in Bipindi in the South (14). The main observation from these data and the present work is that, HIV prevalence in the South region of Cameroon has been stable over time at around 5%. While it is difficult to determine why the prevalence stays high within communities in the South despite widespread ARV used, several factors can be speculated.

The South administrative region of Cameroon has one of the best road networks in the country, connecting it more thoroughly to both other parts of the country, and neighbouring countries (Gabon, Equatorial Guinea and Republic of Congo) than do the road networks of the East administrative region. The higher connectivity of the South relative to the East is expected to favour a greater degree of mixing of human populations and more migration in the South than in the East: a factor which might contribute to lower barriers to HIV-1 spread in the South than in the East (15).

Another potentially important difference between the two regions is the availability and quality of health services for the management of HIV/AIDS. Specifically when the national AIDS control committee assessed health facilities throughout Cameroon in 2018, health facilities in the South ranked lower on average than those anywhere else in the country (15). Factors assessed that were directly relevant to reducing the prevalence of HIV were the accessibility of HIV testing and counselling, the intensity of efforts directed towards prevention of mother-to-child-transmissions, care and support of people living with HIV, and the provision of laboratory and medication management.

We acknowledge that our study has many limitations, including its restriction to two of the five regions of the equatorial rain forest in the country and all have old and mature HIV epidemics. In addition, the lack of individual socio-epidemiological data especially during Period 1 limits the possible impacts that these data may have had on the observed difference in prevalence and in formulating specific recommendations that might help to significantly reduce HIV prevalence in the studied regions.

## Conclusion

In conclusion, our data indicate that HIV-transmission in remote communities of the South administrative region of Cameroon has likely not been curbed over the past decade. The connectivity of this region both to other parts of Cameroon and neighbouring African countries should make this region a priority with respect to containing the spread of HIV and lowering its prevalence. To achieve this though, urgent focused intervention to bolster the quality of HIV-focused healthcare in this region will likely be required; more specifically local authorities should implement and scale-up innovative approaches to HIV testing and provide better linkage to prevention tools and treatment.

## Data Availability

The raw data supporting the conclusions of this article will be made available by the authors, without undue reservation.
